# Clinical correlates of vitamin D deficiency in established psychosis

**DOI:** 10.1186/s12888-016-0780-2

**Published:** 2016-03-22

**Authors:** J. Lally, P. Gardner-Sood, M. Firdosi, C. Iyegbe, B. Stubbs, K. Greenwood, R. Murray, S. Smith, O. Howes, F. Gaughran

**Affiliations:** Department of Psychosis Studies, Institute of Psychiatry, Psychology and Neuroscience (IoPPN), King’s College London, London, SE5 8AF UK; National Psychosis Service, South London and Maudsley NHS Foundation Trust, London, UK; South London and Maudsley NHS Foundation Trust, London, UK; Health Service and Population Research Department, Institute of Psychiatry, Psychology and Neuroscience, King’s College London, London, UK; Physiotherapy Department, South London and Maudsley NHS Foundation Trust, London, UK; Honorary Senior Research Fellow, School of Psychology, University of Sussex, Brighton and Early Intervention in Psychosis Service, Sussex Partnership NHS Foundation Trust, West Sussex, UK; Institute of Psychiatry, Psychology and Neuroscience (IoPPN), King’s College London and Honorary Consultant, National Psychosis Service, South London and Maudsley NHS Foundation Trust, London, UK; Department of Psychosis Studies, MRC Clinical Sciences Centre, Imperial College- Hammersmith Hospital Campus, London, W12 0NN, UK, London, UK

**Keywords:** Schizophrenia, Psychosis, Vitamin D, Metabolic syndrome, Cardiovascular

## Abstract

**Background:**

Suboptimal vitamin D levels have been identified in populations with psychotic disorders.

We sought to explore the relationship between vitamin D deficiency, clinical characteristics and cardiovascular disease risk factors among people with established psychosis.

**Methods:**

Vitamin D levels were measured in 324 community dwelling individuals in England with established psychotic disorders, along with measures of mental health, cardiovascular risk and lifestyle choices. Vitamin D deficiency was defined as serum 25-hydroxyvitamin D (25-OHD) levels below 10 ng/ml (equivalent to <25 nmol/L) and “sufficient” Vitamin D as above 30 ng/ml (>50 nmol/L).

**Results:**

The mean 25-OHD serum level was 12.4 (SD 7.3) ng/ml, (range 4.0-51.7 ng/ml). Forty nine percent (*n* = 158) were vitamin D deficient, with only 14 % (*n* = 45) meeting criteria for sufficiency. Accounting for age, gender, ethnicity and season of sampling, serum 25-OHD levels were negatively correlated with waist circumference (*r* = −0.220, *p* < 0.002), triglycerides (*r* = −0.160, *p* = 0.024), total cholesterol (*r* = −0.144, *p* = 0.043), fasting glucose (*r* = −0.191, *p* = 0.007), HbA1c (*r* = −0.183, *p* = 0.01), and serum CRP levels (*r* = −0.211, *p* = 0.003) and were linked to the presence of metabolic syndrome.

**Conclusions:**

This is the largest cross sectional study of serum 25-OHD levels in community dwelling individuals with established psychosis, indicating a high level of vitamin D deficiency. Lower vitamin D levels are associated with increased cardiovascular disease risk factors and in particular metabolic syndrome. Further research is needed to define appropriate protocols for vitamin D testing and supplementation in practice to see if this can improve cardiovascular disease risk.

**Trial registration:**

ISRCTN number is ISRCTN58667926 Date of registration: 23/04/2010

## Background

Vitamin D levels are sub-optimal in people with schizophrenia and other psychotic disorders [1, 2], being lower than matched controls even from the first episode of psychosis [3]. Lifestyle and physical health factors associated with low vitamin D, such as smoking [4], increased body mass index [5], inactivity, and social withdrawal (likely resulting in deceased sunlight exposure), are all more frequent in people with psychosis.

In the general population, research has demonstrated that individuals with heart failure, hypertension, stroke, and other cardiovascular diseases (CVD) tend to have lower vitamin D levels [6]. Moreover, type 2 diabetes mellitus is more prevalent among people with low vitamin D levels and those who spend less time in the sun [7]. In addition there is an inverse relationship between vitamin D levels and hypertension, obesity and waist circumference [8], all key components of metabolic syndrome (MetS). Recent research has demonstrated that people with psychosis demonstrate high levels of cardiovascular risk, MetS [9, 10] and type 2 diabetes mellitus [11] which are leading contributors to premature mortality in this population [12]. There is accumulating evidence that vitamin D deficiency may be associated with inflammatory cytokines [13] which could explain this relationship. However, to our knowledge, only one other study has investigated this in people with psychosis [14].

Previous studies of vitamin D deficiency in community patients with psychosis have had small sample sizes [15, 16] and few have examined the relationship between vitamin D status and clinical domains of an individual’s illness [15, 17]. In particular, no author has fully explored the relationship between vitamin D and cardiovascular disease risk in people with established psychosis, despite this relationship being observed in the general population [18]. Cardiovascular disease is the leading cause of premature mortality in this population [19], if vitamin D is related to cardiovascular disease risk in people with established psychosis, this may either be amenable to change or be a marker of change, and could assist in collaborative efforts to reduce cardiovascular disease in this population. Importantly, there have been no previous studies reporting the influence of lifestyle factors and duration of time spent outdoors on vitamin D levels in established psychosis.

### Aims

We set out to determine the prevalence of vitamin D deficiency in a cohort of community patients with established psychotic illnesses and ascertain the relationship between vitamin D status and specific clinical features and in particular cardiovascular risk in psychosis.

## Methods

### Study design

This is a cross-sectional study of a random sample of people with established psychosis, recruited at baseline to the NIHR funded Improving Physical health and reducing substance use in Psychosis (IMPaCT) randomised controlled trial (RCT) [20].

### Setting

The study took place within English community mental health teams (CMHTs). Potential participants were identified through their community mental health team care-coordinator. All care-coordinators in participating CMHTs were approached in random sequence and invited to participate. Patients on each participating care coordinator’s caseload, meeting the inclusion criteria, were likewise approached in a random order and sequentially invited to participate.

### Clinical and sociodemographic variables

Sociodemographic and clinical data were collected including gender, age, ethnicity, diagnosis and duration of illness (self-report measure). Diagnoses were based on ICD-10 diagnostic criteria and extracted from the documented diagnosis in the clinical notes at study recruitment. All data were collected at the same timepoint for each individual patient. Participants’ mental health status was measured using the Positive And Negative Syndrome Scale (PANSS) [21], Global Assessment of Functioning (GAF) [22] and the Montgomery Asberg Depression Rating Scale (MADRS) [23]. The EuroQoL-5 dimension (EQ-5D) [24] was used to measure health related quality of life (HRQoL) in participants. Both EQ-5 D index scores and EQ-5 D visual analogue scale scores were recorded. Current smoking was recorded using the Nicotine Dependence Questionnaire [25] and alcohol use by the Alcohol Use Disorders Identification Test (AUDIT) [26]. The International Physical Activity Questionnaire (IPAQ) was used as a measure of physical activity [27]. The IPAQ measures the intensity of physical activity (high, moderate, low) and the duration of walking physical activities over the previous week and is a valid tool for use in this population [28].

All total scores from the scales were derived from the individual scale items. If any of the individual items were missing, then the total score was treated as missing.

### Vitamin D

Levels of Vitamin D (serum 25-hydroxyvitamin D (25-OHD)) were determined by chemiluminescence immunoassay (DiaSorin, S.P.A. Saluggia (Vercelli), Italy). Vitamin D deficiency was defined as 25-OHD levels below 10 ng/ml (equivalent to <25 nmol/L) and vitamin D insufficiency as 25-OHD levels between 10–20 ng/ml (equivalent to 25–50 nmol/L). A serum level of greater than 20 ng/ml (equivalent to >50 nmol/L) is considered optimal [29].

### Cardiovascular risk factors

Anthropometric measures were recorded as described in Gaughran et al., 2013 [20]. Additionally, we measured high sensitivity serum C-reactive protein (HS-CRP) levels. All bloods were fasting. A participant was classified as having a particular cardiovascular risk factor if the measure met the World Health Organisation reference standard for obesity or otherwise met the cut-off used in the diagnosis of metabolic syndrome (MetS) by the International Diabetes Federation [30]. Normal weight, overweight, and obesity therefore were defined as a BMI less than 25 kg/m^2^, 25 to 29.9 kg/m^2^, and 30 kg/m^2^ or higher respectively; central obesity as a waist circumference >94 cm in males and >80 cm in females; hypertension as a systolic BP greater than or equal to 130 mmHg or diastolic greater than or equal to 85 mmHg; dyslipidaemia as having a serum total cholesterol >5 mmol/L; or a LDL >3 mmol/L; and/or an HDL of <1.03 mmol/L in males and <1.29 mmol/L for females; and/or a serum triglyceride level >1.7 mmol/L; type 2 diabetes mellitus (DM) (fasting serum glucose >7.0 mmol/L and/or a HbA1c > 6.5 %).

### Data analysis

Statistical analysis was performed using the IBM Statistical Package for Social Sciences Statistics for Windows, Version 20.0 (Armonk, NY: IBM Corp). Variables were reported as percentages or means ± standard deviation (SD) as appropriate. Data analysis was performed using correlation coefficients, ×^2^-tests, student-t tests and analysis of variance (ANOVA) for parametric data where appropriate.

The primary analysis established the prevalence of vitamin D deficiency in the population. In the secondary analysis we examined the relationships between serum 25-OHD levels and clinical details, including associations between 25-OHD levels and mental health status and with cardiometabolic risk factors, using correlations and ANOVA testing. Partial correlations between serum 25-OHD levels and variables of interest were examined using Pearson’s coefficients controlling for age, gender, ethnicity and season of 25-OHD blood sampling. An ANOVA was conducted to assess for significant differences between mean levels of serum 25-OHD levels within groups. Post hoc analyses using the Tukey post hoc criterion for significance was conducted where ANOVA demonstrated significant differences between the group means. All statistical tests were two-sided and the α- level for statistical significance was 0.05.

### Confounding factors

The following proposed confounding factors were evaluated: gender, age, and ethnicity, smoking status; alcohol use; body mass index. We used the season of blood sampling as a proxy for sunlight irradiation, as it is a factor which will affect the duration and intensity of sunlight exposure. We defined the seasons as follows: summer-June to August; Autumn-September-November; Winter-December to February; Spring–March-May.

Ethical approval for this study was obtained from The Joint South London and Maudsley and The Institute of Psychiatry NHS Research Ethics Committee. Ethical approval was granted on 17^th^ July 2009 (REC Ref no. 09/H080/41). All participants provided informed written consent.

## Results

Vitamin D levels were available for 324 patients (59.6 % male) with a mean age of 43.6 (*SD* = 10.1) years (see Table 1). Participants lived in regions between latitude 52.8 °N and latitude 51.4 °N, with 280 living in urban and 44 in rural settings.

**Table 1:** Clinical/demographic characteristics and differences in mean vitamin D by season of sampling, age categories, ethnicity & diagnoses

	Total (*n* = 324)	Male (*n* = 193)	Female (*n* = 131)				
Mean age (SD)	43.8 (10.1)	43.2 (10.1)	44.7 (10.2)				
Mean Duration of illness (SD)	16.5 (10.4)	17.1 (10.5)	15.6 (10.4)				
Mean Vitamin D levels	12.4 (7.3)	12.2 (7.0)	12.6 (7.8)				
	Mean 25-OHD levels (ng/ml) ± SD				T	df	*P* Value
Age categories	Age ≤ 44 (*n* = 172)		Age > 44 (*n* = 152)				
	12.1 ± 7.1		12.7 ± 7.5		−0.724	322	0.663
Diagnosis	Non affective psychosis (i.e. Schizophrenia) (*n* = 218)		Affective psychosis (Schizoaffective disorder, BPAD, psychotic depression) (*n* = 96)				
	11.5 ± 6.7		14.3 ± 8.1		3.099	312	0.061
	Mean 25-OHD levels (ng/ml) ± SD/Vitamin D deficiency %				F	df	*p* Value
Season of sampling	Autumn (*n* = 96)/38 %	Winter (*n* = 70)/69 %	Spring (*n* = 70)/60 %	Summer (*n* = 88)/36 %	12.977	3	<0.001*
	14.3 ± 7.3^1,2^	9.4 ± 5.9	9.8 ± 4.8^2^	14.7 ± 8.5^1,2^			
Ethnicity	Black (*n* = 104)	White (*n* = 183)	Asian (*n* = 12)	Mixed/Others (*n* = 24)	2.707	3	0.008*
	10.6 ± 5.9^3^	13.5 ± 8.1^3^	10.7 ± 8.4	12.4 ± 4.9			

Student-t tests and analysis of variance (ANOVA) were conducted to assess for differences in serum 25-OHD between groups. The *values represent the significant difference with *p* <0.05

^1,2^Mean serum 25-hydroxyvitamin D (25-OHD) levels were significantly decreased in those who had blood sampling in Winter compared to Summer or Autumn (*p* < 0.001) and were significantly decreased in Spring compared to Summer or Autumn (*p* < 0.001)

^3^Mean 25-OHD levels were significantly decreased in those of black ethnicity compared to those of white ethnicity (*p* = 0.002)

### Vitamin D levels and deficiency

The mean 25-OHD serum level was 12.4 (SD 7.3) ng/ml (range 4.0−51.7 ng/ml). Almost half of the sample (48.8 %, *n* = 158) were deficient in vitamin D while only 13.9 % (*n* = 45) had sufficient vitamin D.

### Vitamin D levels and clinical and demographic characteristics

The associations between 25-OHD levels and demographic, and clinical features are shown in Table 1. Briefly, mean 25-OHD levels were significantly lower in those of black African/black Caribbean ethnicity than in those of white ethnicity. Serum 25-OHD levels did not differ between males (mean 12.2 ng/ml (*SD* = 7.0) and females (mean 12.6 ng/ml (*SD* = 7.8) (*t* = −0.511, *p* = 0.610)); nor between urban (mean 12.4 (SD 7.5) ng/ml and rural settings (mean 12.5 (SD 6.0) (*p* = 0.907). There was no significant relationship between serum 25-OHD levels and duration of illness (*r* = −0.015, *p* = 0.808).

### Vitamin D levels and mental state

Serum 25-OHD levels were not significantly associated with PANNS total scores (*r* = −0.106, *p* = 0.058); PANSS positive scores (*r* = −062, *p* = 0.272); PANSS negative scores (*r* = −0.103, *p* = 0.066); GAF score (*r* = −062, *p* = 0.270); or MADRS score (*r* = −087, *p* = 0.117).

### Vitamin D levels and cardiovascular disease risk factors

Associations between cardiovascular risk factors and serum 25-OHD levels are shown in Table 2. After adjustment for age, gender and ethnicity and season of sampling, serum 25-OHD levels were significantly negatively correlated with waist circumference (*r* = −0.220, *p* < 0.002), BMI (*r* = −0.163, *p* = 0.022), triglycerides (*r* = −0.160, *p* = 0.024), total cholesterol (*r* = −0.144, *p* = 0.043), fasting glucose (*r* = −0.191, *p* = 0.007), HbA1c (*r* = −0.183, *p* = 0.01), and CRP (*r* = −0.211, *p* = 0.003). Those with vitamin D deficiency (serum 25-OHD levels <10 ng/ml) had mean CRP levels of 7.8 (SD 10.4) mg/L, significantly higher than those who were not vitamin D deficient (serum CRP of 3.9 (SD 4.3) mg/L (*t* = 3.789, *p* < 0.001). Further serum 25-OHD levels were significantly negatively correlated with hypertension (*r* = −0.180, *p* = 0.011).

**Table 2:** Significant bivariate and partial correlations between serum levels of 25-Hydroxyvitamin D (25-OHD) and cardiometabolic risk factors among patients with psychosis

	Bivariate r for 25-OHD level	Partial r for 25-OHD level
Waist	−0.188**	−0.220**
BMI	−0.149*	−0.163*
Type 2 DM	−0.147*	−0.128
MetS	−0.117*	−0.151*
Fasting glucose	−0.135*	−0.191**
HbA1c	−0.146**	−0.183**
Serum triglyceride	−0.133*	−0.160*
Serum total cholesterol	−0.128*	−0.144*
Systolic blood pressure	−0.084	−0.134
Diastolic blood pressure	−0.151**	−0.128
CRP level	−0.206**	−0.211**

Bivariate and partial correlations (r) between serum 25-OHD levels and cardiometabolic risk factors were examined using Pearson’s coefficients, with controlling for age, gender, ethnicity and season of 25-OHD blood sampling. The *values represent the significant difference with *p* <0.05, **with *p* < 0.01 and ***with *p* < 0.001

**Fig. 1:**
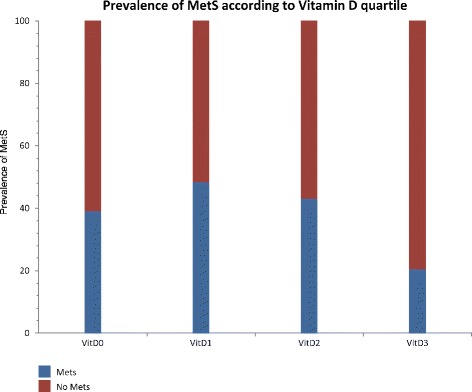
Prevalence of Metabolic Syndrome (MetS) according to Vitamin D quartile. footnote: First (*VitD0*), second (*VitD1*), third (*VitD2*) and fourth (*VitD3*) quartiles with mean serum 25-OHD levels of 5.4 ng/ml (*SD* = 1.0), 8.6 ng/ml (*SD* = 0.94), 12.8 ng/ml (*SD* = 1.8), and 22.8 ng/ml (*SD* = 6.3), and 25-OHD levels in each of *VitD0*, *VitD1*, *VitD2* and *VitD3* which ranged from 34.0−7.0 ng/ml, 7.0−10.2 ng/ml, 10.3−16.4 ng/ml and 16.5−51.7 ng/ml. Logistic regression was performed to test the differences

The prevalence of metabolic syndrome (as defined by the International Diabetes Federation [30]) in association with increasing quartiles of serum 25-OHD was investigated. Participants within the highest quartile of vitamin D (mean serum 25-OHD level 22.8 ng/ml (*SD* = 6.3; range 16.5−51.7 ng/ml) had the lowest prevalence of MetS (*N* = 73, 20.5 %) (see Fig. 1). Participants within the first (*N* = 92, 39.1 % MetS), second (*N* = 93, 48.3 % MetS) and third (*N* = 95, 43.15 % MetS) lowest vitamin D quartiles were at 2.48 (OR 95 % CI 1.22 to 5.03), 3.62 (OR 95 % CI 1.80 to 7.28), 2.93 (OR 95 % CI 1.46 to 5.89) greater odds of having MetS compared to participants within the highest quartile of vitamin D.

### Vitamin D levels and lifestyle factors

There was no significant difference between serum 25-OHD levels in current tobacco smokers (*n* = 197) versus non-smokers (*n* = 127) (*p* = 0.821) nor any association between serum 25-OHD levels and alcohol use as measured by the AUDIT (*p* = 0.455). The use of vitamin supplements (*n* = 20) did not affect 25-OHD levels (*t* = −0.712, *p* = 0.477).

Those engaging in low intensity physical activity over the week prior to sampling (*n* = 137) had significantly lower 25-OHD levels (mean =10.91 ng/ml (*SD* = 6.87) than those who engaged in moderate or high intensity physical activity (*n* = 187) (mea*n* = 13.45 ng/ml (*SD* = 7.47) (*t* = −3.123, *df* =322, *p* = 0.002). There was a direct relationship between serum 25-OHD levels and the amount of hours spent outdoors (*r* = 0.140, *p* = 0.012).

## Discussion

To our knowledge, this is the largest study to date investigating 25-OHD levels in community dwelling individuals with established psychosis. Nearly half of our study sample had vitamin D deficiency, more than in previously published studies of psychosis, for example the 26 % (*n* = 34) seen in a Norwegian study of outpatients with psychosis [15]. As in the general population, black African or black Caribbean people with psychosis had lower levels than their white counterparts. Moreover, we have for the first time in a population with established psychosis, identified that those with the highest levels of vitamin D have a lower prevalence of MetS (20.5 %), compared to those in the lowest (39.1 %), second (48.3 %) and third quartile (43.1 %) of vitamin D (all *p* < 0.01). For the first time we have demonstrated that those people with psychosis that engage in more moderate and vigorous physical activity have significantly higher levels of vitamin D (*p* = 0.002).

### Vitamin D and cardiometabolic risk

Perhaps the most novel and significant finding within this study is the relationships we observed between 25-OHD levels and cardiovascular risk factors in psychotic illnesses. We observed a clear inverse relationship between rising vitamin D levels and decreased MetS risk, which has important implications given that MetS and cardiovascular disease are highly prevalent [9, 10] and leading causes of premature mortality [19]. Of interest, we identified associations with hypertension and low 25-OH levels. This is likely to be clinically important as in the general population low 25-OHD levels are associated with the risk of incident CVD and specifically hypertension [31]. Further, the inverse relationship between 25-OHD levels and dyslipidaemia, previously demonstrated in large general population samples, and replicated here, is worthy of further exploration given the high risk of cardiovascular morbidity in psychosis. The strongest correlations with low 25-OHD levels were with factors related to high body fat. This is consistent with findings that those with increased adipose tissue stores (in which vitamin D, being fat soluble, is stored), due to obesity, have lower circulating vitamin D due to this increased storage capacity [32]. The low 25-OHD levels could then be a secondary manifestation of obesity.

Given these results, the questions that one could pose is, would the supplementation of vitamin D in psychosis prevent and/or ameliorate cardiovascular and metabolic risk? This is a key question that needs addressing in future work.

Raised levels of the inflammatory marker CRP, which is pathogenic in the development of CVD, have been described in both schizophrenia [33] and bipolar affective disorder [34]. This is the largest study to identify an association between raised CRP and vitamin D deficiency in established psychosis, with a similar inverse relationship between CRP and serum 25-OHD having been previously identified in schizophrenia patients in a case–control study [14]. This is interesting in the context of vitamin D’s known anti-inflammatory properties, especially given the recent resurgence of research into auto-immunity and inflammation in psychosis. Clinical trials have shown inconsistent results regarding the benefits of vitamin D supplementation in reducing CRP levels in the general population [35], but this may be worth further examination in psychosis.

Another novel finding from this study is the association between low vitamin D in established psychotic disorders and decreased physical activity [15]. This further adds to the notion that engaging people with psychosis may improve health outcomes, including vitamin D status. The association between time spent outside and higher serum 25-OHD levels provides support for selective strategies to encourage increased outdoor activity in people with psychosis –not only to boost vitamin D levels but for all the other known health benefits. Vitamin D levels were not appreciably higher in those taking vitamin D supplements. This may support the notion that most of these supplements contain much too low a dose of vitamin D to have a meaningful effect on serum levels [36], although there remains the possibility, not tested, that supplements may have been prescribed because of an already suspected inadequate level or that treatment non-adherence may have occurred.

The lack of a prospective study design means further work is needed to answer the question of causality; however, in the meantime it is important that primary and secondary health care professionals are aware of the high rates of vitamin D deficiency in people with established psychosis. This is particularly so, given that people with psychotic illnesses residing in the geographical area where this work was carried out, have a life span shortened by up to 18 years [37]. This reduced life expectancy is largely due to cardiovascular disease, for which vitamin D deficiency is a known risk factor in the general population [31, 18]. It is also clinically noteworthy that vitamin D deficiency may exacerbate the effects of lower bone mineral density and increased osteoporosis seen in psychosis [38, 39], particularly as osteoporosis is more prevalent in black patients [40]. This may partially account for the fact that people with schizophrenia have more fractures than the general population [41].

### Comparison with other populations

Studies of inpatients with schizophrenia have reported vitamin D deficiency rates of 11 %–26 % [1, 2]. A mean 25-OHD level of 29.0 ng/ml was observed in one inpatient sample of 34 schizophrenia patients, much higher than that in our UK sample [42]. Our vitamin D deficiency prevalence of 50 % contrasts with that of an epidemiological cohort (*N* =7437) of UK males, where 16 % had vitamin D deficiency (using the same definitions) in the winter and spring (64 % in our study), falling to 3 % in the summer and autumn (36 % in our study) [43]. In the 2004 UK National Health Survey, 14 % of men and 15 % of women were vitamin D deficient (using the same definitions) compared to of 48 % of males and 51 % of females in our study. This excess is highlighted when an aged-matched comparison is used; for those aged 35–49 in the same study, the mean 25-OHD level was 48 nmol/L (equivalent to 19.2 ng/ml), compared to the mean 25-OHD level of 12.1 ng/ml in this age group in our study [44].

Similar to findings from a recent mini meta-analysis, [45] we also observed that individuals with schizophrenia had a trend towards lower 25-OHD levels than those with other psychotic disorders. However, since our numbers with diagnoses of schizoaffective disorder and bipolar affective disorder are relatively small, it is difficult to draw firm conclusions. We did not identify significant correlations with clinical symptoms and 25-OHD levels, but an inverse relationship between 25-OHD levels and psychotic and depressive symptoms is the overall trend in these analyses in keeping with expectations.

### Strengths and limitations

The study is limited by its cross sectional design, meaning that no inference on the directionality of the association between vitamin D and psychotic disorders or symptoms can be made. As such further replication in large prospective cohorts would be valuable. We have identified a higher prevalence of vitamin D deficiency in established psychosis when compared to general population samples, though comparisons are limited by an inability to control for ethnicity and by the use of different assay techniques. The use of automated immunoassays to quantify 25-OHD levels may limit the generalisability of the findings due to variable concordance between different mechanisms of 25-OHD measurement. In particular, immunoassays may underestimate true vitamin D deficiency, as they can overestimate serum 25-OHD results at serum levels <8 ng/ml (<20 nmol/L) [46], which could have potentially led to an underestimate of vitamin D deficiency in this sample. Study strengths include the large sample size, random sampling across multiple sites, clinical diagnoses of schizophrenia and other psychotic disorders, measures of clinical status and the multi-ethnicity of the population, which should enhance the study’s generalisability.

## Conclusions

Because of the potential adverse effects of vitamin D deficiency on the skeletal system and on cardiovascular morbidity, the question of whether to recommend widespread screening for vitamin D deficiency or routine vitamin D supplementation arises. There is as yet no evidence that Vitamin D supplementation will improve psychotic symptoms, so the question should be looked at in the light of general population guidelines. Evidence is lacking here too, pending the results of large ongoing RCTs [47] and clinical equipoise remains about the general health benefits of routine population screening and supplementation of vitamin D [48]. However, given the extremely low levels of vitamin D seen in this vulnerable population, large randomised trials, specifically in psychosis, are urgently needed to determine whether supplementation with vitamin D is an effective means of ameliorating mental health outcomes [49], boosting cardiometabolic health and ultimately reducing mortality in psychosis.

### Ethics approval and consent to participate

Ethical approval for this study was obtained from The Joint South London and Maudsley and The Institute of Psychiatry NHS Research Ethics Committee. Ethical approval was granted on 17th July 2009 (REC Ref no. 09/H080/41). All participants provided informed written consent.

### Availability of data and materials

The data cannot be shared due to confidentiality issues, though individual data requests can be considered.

## Abbreviations

25-OHD: 25-hydroxyvitamin D
ANOVA: analysis of variance
AUDIT: alcohol use disorders identification test
CMHTs: community mental health teams
CVD: cardiovascular diseases
DM: diabetes mellitus
EQ-5D: EuroQoL-5 dimension
GAF: global assessment of functioning
HS-CRP: high sensitivity serum C-reactive protein
IPAQ: international physical activity questionnaire
MADRS: montgomery asberg depression rating scale
MetS: metabolic syndrome
PANSS: positive and negative syndrome scale
RCT: randomised controlled trial
